# Nonnegligible Seroprevalence and Predictors of Murine Typhus, Japan

**DOI:** 10.3201/eid2907.230037

**Published:** 2023-07

**Authors:** Tetsuro Aita, Eiichiro Sando, Shungo Katoh, Sugihiro Hamaguchi, Hiromi Fujita, Noriaki Kurita

**Affiliations:** Fukushima Medical University, Fukushima, Japan (T. Aita, E. Sando, S. Katoh, S. Hamaguchi, N. Kurita);; Kita-Fukushima Medical Center, Fukushima (E. Sando, S. Katoh, H. Fujita)

**Keywords:** murine typhus, seroepidemiological studies, cross-sectional studies, *Rickettsia typhi*, typhus, flea borne, scrub typhus, *Orientia tsutsugamushi*, Japanese spotted fever, *Rickettsia japonica*, spotted fever group rickettsiosis, rickettsia, vector-borne infections, Japan, bacteria

## Abstract

To elucidate the epidemiology of murine typhus, which is infrequently reported in Japan, we conducted a cross-sectional study involving 2,382 residents of rickettsiosis-endemic areas in Honshu Island during August–November 2020. *Rickettsia typhi* seroprevalence rate was higher than that of *Orientia tsutsugamushi*, indicating that murine typhus is a neglected disease.

Murine typhus (MT), a fleaborne rickettsiosis caused by the bacterium *Rickettsia typhi*, is a ubiquitous but clinically less recognizable disease than scrub typhus or spotted fever group rickettsioses (*1*). Limited testing because of the infection’s nonspecific symptoms and the need for expert laboratories for serodiagnosis makes MT an underrecognized infection. MT occurs worldwide and is endemic to warm urban or coastal regions where the climate is favorable for rats, which can serve as the reservoir of *R. typhi*. However, epidemiologic characteristics and risk factors often vary by region (*1*–*3*). Therefore, accumulating specific and local evidence from each region is required to elucidate the complete picture of MT epidemiology.

In Japan, MT with *Xenopsylla cheopis* fleas as the vector and *Rattus rattus* or *R. norvegicus* rats as the reservoir was endemic before the 1950s (*4*), but the disease has not been notifiable; only a few cases have been reported since then (*5*). As a consequence, the epidemiologic characteristics remain unknown, rendering MT an underrecognized and neglected infection. Clarifying the epidemiologic features of MT in Japan will help clinicians recognize the disease and provide early treatment. We estimate the seroprevalence of rickettsia, primarily of *R. typhi*, in rickettsia-endemic areas of Honshu Island (the largest island of Japan) and characterize the risk factors for MT.

## The Study

We conducted a cross-sectional study in 3 sites in the southeastern part of Honshu (Boso Peninsula), endemic areas for scrub typhus and Japanese spotted fever (*6*). We included persons who underwent regular checkups during August–November 2020 (Appendix). Questionnaires were distributed during checkups, and the following data were collected: medical history of rickettsioses; environmental exposure to mountains, agriculture, and bushes; and residential addresses. The respondents were asked through questionnaires whether they resided in or had visited mountainous areas, had visited areas with small trees and weeds, or engaged in agricultural work. In addition, we measured the population density and area of each land use (coasts, forests, farmland, rivers or lakes, and wilderness) within a 500-meter radius of the participant’s address (Appendix). The study was approved by the Institutional Review Boards of Nagasaki University and Fukushima Medical University (approval nos. 200305230-2 and 2022-190). Written consent was obtained from all participants.

The primary outcomes were *R. typhi* seroprevalence and ratio of *R. typhi to Orientia tsutsugamushi* seroprevalence. *O. tsutsugamushi* was selected as the comparator outcome because scrub typhus is a notifiable disease and the rickettsiosis most endemic to Japan. Furthermore, we evaluated the seroprevalence of *R. japonica*, the pathogen of Japanese spotted fever, to determine the possibility of an apparently high seroprevalence of *R. typhi* because of cross-reactivity in the genus *Rickettsia* (*7*) (Appendix). We defined seropositivity as a ratio of >1:40 and defined *O. tsutsugamushi* seropositivity as a positive result for any of the *O. tsutsugamushi* serotypes. The sensitivity analyses estimated the seroprevalences at cutoff titers of 1:80 and 1:160.

Because the seropositivity rates of *R. typhi* and *O. tsutsugamushi* were regarded as paired binomial data, we tested the difference in their prevalence by using the McNemar test (*8*) and estimated it using conditional Poisson regression (Appendix). To explore the factors associated with *R. typhi* seropositivity, we fitted a logistic regression model by using the candidate risk factors. We assessed whether there were differences in the seroprevalence ratios across study sites and conducted the imputation of missing values (Appendix).

The median age of all participants was 67 years. *R. typhi*–seropositive participants exhibited a lower population density than *R. typhi*–seronegative participants, showing a similar trend to *O. tsutsugamushi* (Table 1; Appendix Table 1). The residential locations of *R. typhi*–seropositive participants were distributed throughout the Boso Peninsula, and a similar distribution was observed for *O. tsutsugamushi*–seropositive participants (Appendix Figures 1, 2). Although ≈60% of *R. typhi*–seropositive participants had titers of <160, 20 participants had titers of >1,280, and 4 had titers of >40,960 (Table 2). Most *O. tsutsugamushi*–seropositive participants had lower titers, although some exhibited notably high titers (Appendix Table 2).

**Table 1:** Characteristics and residential geographic features for participants in study of seroprevalence and predictors of murine typhus, by *Rickettsia typhi* IgG seropositivity status, Japan*

Characteristic	*R. typhi*–positive, n = 269	*R. typhi*–negative, n = 2,113	Total, n = 2,382
Sex			
F	123 (45.7)	1,080 (51.1)	1,203 (50.5)
M	146 (54.3)	1,033 (48.9)	1,179 (49.5)
Age group, y			
<40	2 (0.7)	109 (5.2)	111 (4.6)
41–50	11 (4.1)	269 (12.7)	280 (11.8)
51–60	15 (5.6)	350 (16.6)	365 (15.3)
61–70	71 (26.4)	695 (32.9)	766 (32.2)
71–80	113 (42)	576 (27.2)	689 (28.9)
>81	57 (21.2)	114 (5.4)	171 (7.2)
Site			
Otaki	180 (66.9)	891 (42.2)	1,071 (45)
Katsuura	42 (15.6)	250 (11.8)	292 (12.2)
Kameda	47 (17.5)	972 (46)	1,019 (42.8)
Medical history			
None	227 (84.4)	1,787 (85)	2,014 (84.9)
Scrub typhus	8 (3.0)	32 (1.5)	40 (1.7)
Japanese spotted fever	2 (0.7)	0	2 (0.1)
Both	0	1 (0.1)	1 (0)
Unknown	32 (11.9)	283 (13.5)	315 (13.3)
Missing	0	10	10
Environmental exposure history			
Mountains			
Yes	70 (26)	456 (21.6)	526 (22.1)
No	199 (74)	1,657 (78.4)	1,856 (77.9)
Agriculture			
Yes	135 (50.2)	766 (36.3)	901 (37.8)
No	134 (49.8)	1,347 (63.7)	1,481 (62.2)
Bushes†			
Yes	141 (52.4)	856 (40.5)	997 (41.9)
No	128 (47.6)	1,257 (59.5)	1,385 (58.1)
Environment surrounding the residence			
Population density, persons/km^2^ (5th–95th percentile)	244 (32–1,207)	356 (45–3,354)	306 (44–3,148)
Missing	3	26	29
Coasts, m^2^ (5th–95th percentile)	0 (0–235,132)	0 (0–232,102)	0 (0–233,502)
Missing	0	3	3
Forests, m^2^ (5th–95th percentile)	307,376 (76,276–664,633)	269,342 (6,023–616,031)	273,757 (7,231–621,401)
Missing	0	3	3
Farmland, m^2^ (5th–95th percentile)	236,624 (14,833–458,114)	233,350 (0–486,875)	233,414 (0–483,051)
Missing	0	3	3
Rivers and lakes, m^2^ (5th–95th percentile)	17,596 (0–101,103)	13,917 (0–84,788)	14,279 (0–86,124)
Missing	0	3	3
Wilderness, m^2^ (5th–95th percentile)	0 (0–27,181)	0 (0–25,095)	0 (0–25,391)
Missing	0	3	3

*Values are no. (%) except as indicated. Continuous and categorical variable data are presented as median (5th–95th percentile) and frequency (%). †Bushes refer to areas with small trees and weeds.

**Figure 1:**
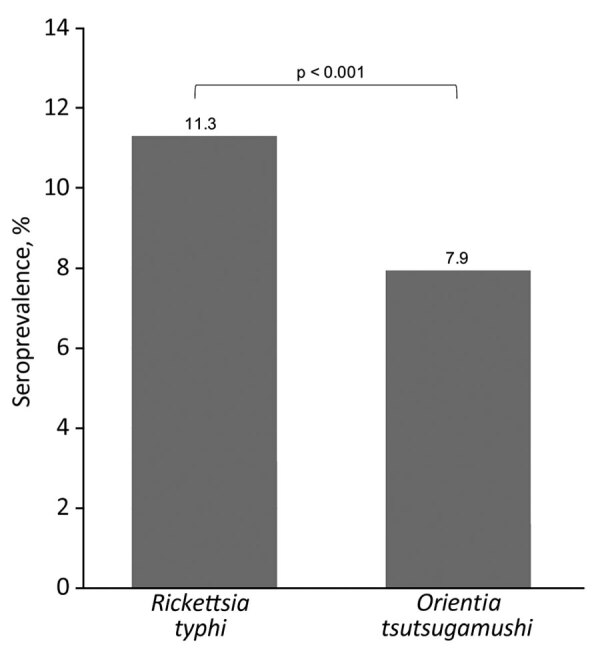
Seroprevalence rates of *Rickettsia typhi* and *Orientia tsutsugamushi* in study of seroprevalence and predictors of murine typhus, Japan. *R. typhi* IgG was detected in 11.3% (95% CI 10.0–12.6) of participants and *O. tsutsugamushi* IgG was detected in 7.9% (95% CI 6.9–9.1) of all participants. The seroprevalence of both infections was compared using the McNemar test. The estimated seropositivity ratio was 1.42 (95% CI 1.20–1.68).

**Figure 2:**
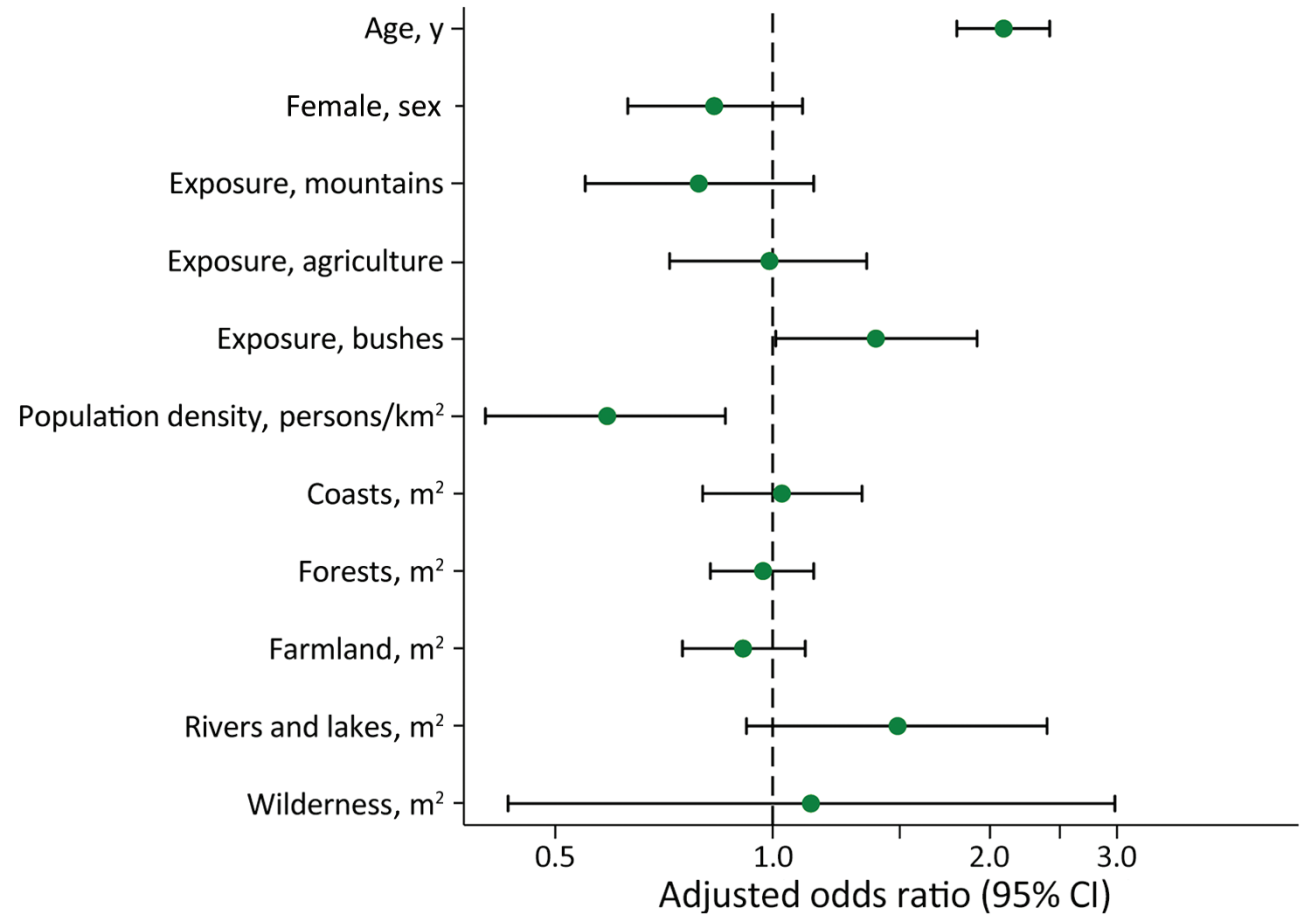
Predictors of *Rickettsia typhi* IgG seropositivity in study of seroprevalence and predictors of murine typhus, Japan. Shown are adjusted odds ratios for age per 10-year increase; population density increase; residential geographic features, such as coasts, forests, farmland, and rivers and lakes; and wilderness per 10-hectare increase. Bushes refer to areas with small trees and weeds.

**Table 2:** Distribution of antibody titers in *Rickettsia typhi* IgG–positive persons in study of seroprevalence and predictors of murine typhus, Japan

Antibody titer	Seropositive participants, no. (%), n = 269
1:40	53 (19.7)
1:80	33 (12.3)
1:160	80 (29.7)
1:320	37 (13.7)
1:640	46 (17.1)
1:1,280	5 (1.9)
1:2,560	8 (3.0)
1:5,120	2 (0.7)
1:10,240	1 (0.4)
1:20,480	0
>1:40,960	4 (1.5)

*R. typhi* seroprevalence was 11.3% higher than that of *O. tsutsugamushi* (7.9%) (ratio of seropositivity 1.42; 95% CI 1.20–1.68) (Figure 1). Of the 2,382 residents, 204 were *R. japonica* seropositive, for a seroprevalence of 8.6%, lower than that of *R. typhi*. Furthermore, the antibody titer for *R. typhi* was higher than that for *R. japonica* in participants who were seropositive for both *R. typhi* and *R. japonica* (Appendix Table 3). Results of the sensitivity analyses did not show any changes to the predominance of the *R. typhi* seroprevalence over the *O. tsutsugamushi* seroprevalence (Appendix Table 4). 

According to the multivariate analysis (Figure 2), the factors associated with *R. typhi* seropositivity were age (per 10-year increase; adjusted odds ratio [aOR] 2.09 [95% CI 1.80–2.42]), low population density (per 1,000 persons/km^2^ increase; aOR 0.59 [95% CI 0.40–0.86]), and history of bush exposure (aOR 1.39 [95% CI 1.01–1.92]) (Appendix).

## Conclusions

We demonstrated robust findings of the predominance of *R. typhi* seroprevalence over *O. tsutsugamushi* seroprevalence in rickettsia-endemic areas in Japan. Previously, in those study areas, an epidemiologic study was conducted using now antiquated serologic methods, such as the Weil–Felix test (*4*), which provided unreliable MT and scrub typhus seroepidemiologic data (*9*). Contrary to the previous study’s findings, we were able to estimate rickettsiosis seroprevalence and confirm the predominance of MT more precisely using the standard diagnostic test.

This study illustrated that MT is a prevalent and possibly reemerging infection in Japan. Recently, in the United States (*10*), Greece (*11*), and Spain (*12*), the incidence of MT has increased, partly because of improved disease recognition (*10*) and a change in the transmission route (*13*). Thus, given the high seroprevalence of *R. typhi* in Japan, case accumulation is crucial to clarify the possibility of a unique transmission cycle.

The risk factors for *R. typhi* seropositivity in this study differed from those in previous studies. The increase in *R. typhi* seroprevalence with decrease in residential population density contradicts the findings of previous studies that showed urban environment as a risk factor (*2*,*14*). In addition, exposure to weeded areas was identified as a risk factor, but residential environments, including those near coasts, rivers, and lakes, which have been reported as risk factors (*2*,*3*), were not correlated. The differences in risk factors between this study and previous studies might reflect differences in factors related to contact with vectors and reservoirs at each study site.

The first limitation of our study is that seropositivity to *R. typhi* could indicate cross-reactivity to *R. japonica*. However, because *R. typhi* seropositivity was higher than *R. japonica* seropositivity and the cross-reactivity rate to *R. typhi* in confirmed Japanese spotted fever patients is ≈20% (*15*), most patients with *R. typhi* seropositivity have a true MT infection. Second, we did not consider cross-reactivity within the same group (spotted fever or typhus group) in the genus *Rickettsia*. However, other diseases caused by this genus have been reported infrequently, except for Japanese spotted fever and MT in domestic infection cases. Third, this study was conducted in persons undergoing routine checkups and might not represent seroprevalence in the general population.

In summary, *R. typhi* seroprevalence was higher than that of *O. tsutsugamushi* in rickettsia-endemic areas of Japan, indicating that MT is a neglected and underrecognized condition. This study highlights the need to include MT in the differential diagnosis when examining patients with nonspecific infectious symptoms who are residing in rickettsia-endemic areas. Clinicians should consider comprehensive examinations for rickettsial infections, including MT testing, especially in those with a history of residence in sparsely populated areas or exposure to bushes.

Appendix Additional information about nonnegligible seroprevalence and predictors of murine typhus, Japan

## References

[1] Civen R, Ngo V. Murine typhus: an unrecognized suburban vectorborne disease. Clin Infect Dis. 2008;46:913–8. 10.1086/527443 18260783

[2] Azad AF. Epidemiology of murine typhus. Annu Rev Entomol. 1990;35:553–69. 10.1146/annurev.en.35.010190.003005 2105686

[3] Devamani CS, Schmidt WP, Ariyoshi K, Anitha A, Kalaimani S, Prakash JAJ. Risk factors for scrub typhus, murine typhus, and spotted fever seropositivity in urban areas, rural plains, and peri-forest hill villages in South India: a cross-sectional study. Am J Trop Med Hyg. 2020;103:238–48. 10.4269/ajtmh.19-0642 32458785 PMC7356468

[4] Tamiya T. Recent advances in studies of tsutsugamushi disease in Japan. Tokyo: Medical Culture, Inc.; 1962. p. 53–54.

[5] Sakaguchi S, Sato I, Muguruma H, Kawano H, Kusuhara Y, Yano S, et al. Reemerging murine typhus, Japan. Emerg Infect Dis. 2004;10:964–5. 10.3201/eid1005.030697 15216852 PMC3323209

[6] Sando E, Suzuki M, Katoh S, Fujita H, Taira M, Yaegashi M, et al. Distinguishing Japanese spotted fever and scrub typhus. Emerg Infect Dis. 2018;24:1633–41. 10.3201/eid2409.171436 30124190 PMC6106405

[7] Uchiyama T, Zhao L, Yan Y, Uchida T. Cross-reactivity of *Rickettsia japonica* and *Rickettsia typhi* demonstrated by immunofluorescence and Western immunoblotting. Microbiol Immunol. 1995;39:951–7. 10.1111/j.1348-0421.1995.tb03298.x 8789054

[8] Pembury Smith MQR, Ruxton GD. Effective use of the McNemar test. Behav Ecol Sociobiol. 2020;74:133. 10.1007/s00265-020-02916-y

[9] Stewart AG, Stewart AGA. An update on the laboratory diagnosis of rickettsia spp. infection. Pathogens. 2021;10:1319. 10.3390/pathogens10101319 34684267 PMC8541673

[10] Ruiz K, Valcin R, Keiser P, Blanton LS. Rise in murine typhus in Galveston County, Texas, USA, 2018. Emerg Infect Dis. 2020;26:1044–6. 10.3201/eid2605.191505 32310080 PMC7181902

[11] Labropoulou S, Charvalos E, Chatzipanagiotou S, Ioannidis A, Sylignakis P, Τaka S, et al. Sunbathing, a possible risk factor of murine typhus infection in Greece. PLoS Negl Trop Dis. 2021;15:e0009186. 10.1371/journal.pntd.0009186 33711035 PMC7990230

[12] Rodríguez-Alonso B, Almeida H, Alonso-Sardón M, Velasco-Tirado V, Robaina Bordón JM, Carranza Rodríguez C, et al. Murine typhus. How does it affect us in the 21st century? The epidemiology of inpatients in Spain (1997-2015). Int J Infect Dis. 2020;96:165–71. 10.1016/j.ijid.2020.04.054 32353550

[13] Penicks A, Krueger L, Morgan T, Nguyen K, Campbell J, Fogarty C, et al. Jumping into the future: an analysis of 50 years of flea data from mammalian wildlife collected during three flea-borne rickettsioses surveys in Orange County, 1967–2017. Proc Pap Annu Conf Mosq Vector Control Assoc Calif. 2019;87:1.

[14] Yao Z, Tang J, Zhan FB. Detection of arbitrarily-shaped clusters using a neighbor-expanding approach: a case study on murine typhus in south Texas. Int J Health Geogr. 2011;10:23. 10.1186/1476-072X-10-23 21453514 PMC3079590

[15] Aita T, Sando E, Katoh S, Hamaguchi S, Fujita H, Kurita N. Serological cross-reactivity between spotted fever and typhus groups of rickettsia infection in Japan. Int J Infect Dis. 2023;130:178–81. 10.1016/j.ijid.2023.03.012 36907548

