# Supplementary material for: Nonnegligible Seroprevalence and Predictors of Murine Typhus, Japan
Source: Emerg Infect Dis. 2023 Jul;29(7):1438–42. doi: 10.3201/eid2907.230037 (PMC10310371; doi:10.3201/eid2907.230037)
Supplement: Appendix — Additional information about nonnegligible seroprevalence and predictors of murine typhus, Japan [file 23-0037-Techapp-s1.pdf]

Article DOI: https://doi.org/10.3201/eid2907.230037

EID cannot ensure accessibility for supplementary materials supplied by authors. Readers who have difficulty accessing supplementary content should contact the authors for assistance.

Nonnegligible Seroprevalence and Predictors of Murine Typhus, Japan

Appendix

Methods

Study sites

This study was conducted in two local municipalities (Otaki Town and Katsuura City) and Kameda Medical Center (Kamogawa City). According to the 2015 census, Otaki and Katsuura have a total population of 9,843 and 19,248, respectively (https://www.e-stat.go.jp/). Kameda Medical Center is a tertiary hospital with 865 acute beds allowed for patients from all over the prefecture. We chose multiple sites for our study because rickettsioses, including scrub typhus and Japanese spotted fever, are endemic at these sites and are reported annually (*1*) and epidemiologic studies have been conducted previously (*2*). In addition, we wanted to ensure a diversity of participants.

Health checkups

The health checkup services in Otaki and Katsuura were provided mainly to the National Health Insurance subscribers, whereas the checkup services in Kameda Medical Center were offered to anyone who desired it, regardless of the individual's insurance category.

Measurement of population density and the area of each land use

Population density and land use area per 1 km^2, as registered in the 2015 national data (https://nlftp.mlit.go.jp/index.html), were linked to each participant's address to obtain the population density and the area of each land use within a 500-meter radius of the participant's address, using QGIS 3.16 (https://qgis.org/ja/site/).

Collection of blood samples and measurement of rickettsial antibody levels

After obtaining the participants' consent, the levels of serum rickettsial antibodies were measured using residual serum (0.5 mL) from blood samples of participants who underwent health checkups. The samples were frozen at −20°C and sent to the Mahara Institute of Medical Acarology (Anan, Japan) for indirect immunoperoxidase assay to measure the IgG antibody levels of the six serotypes of *Orientia tsutsugamushi* (Kato, Karp, Gilliam, Irie/Kawasaki, Hirano/Kuroki, and Shimokoshi), *Rickettsia japonica* (Aoki strain), and *Rickettsia typhi* (Wilmington strain) (*3*,*4*). These samples were diluted from 1:40 to 1:40,960.

Statistical analyses

All statistical analyses were performed using Stata 17.0. To estimate the magnitude of the difference in seropositivity between the two different antibody assays (i.e., *R. typhi* and *O. tsutsugamushi*) (*5*), conditional Poisson regression with a robust variance estimator was used (*6*,*7*). To test whether the magnitude of the prevalence ratios differed across the study sites, an interaction between the pair and site variables was added to the regression model (*5*). The Wald test was used to examine the interactions. Owing to the missing values for population density and environmental exposure, multiple imputation was performed assuming that the missing values occurred at random (*8*). For this study, five complete datasets were generated using multiple imputation with chained equations. The odds ratios obtained from the imputed data were combined according to the Rubin's rule. A two-sided p value of <0.05 was considered significant.

Results

Estimated prevalence ratios by study site

The estimated prevalence ratios of *R. typhi* to *O. tsutsugamushi* in the aforementioned three sites based on the conditional Poisson regression analysis results were 1.63 (95% confidence interval [CI]: 1.32–2.00), 1.05 (95% CI: 0.73–1.50), and 1.23 (95% CI: 0.79–1.92) in Otaki, Katsuura, and Kameda, respectively. However, the Wald test for the study-site difference in the seroprevalence ratio showed a p value of 0.093, indicating that the null hypothesis of no study-site difference in the seropositivity ratio between *R. typhi* and *O. tsutsugamushi* could not be rejected.

Non-predictors of *Rickettsia typhi* seropositivity

The extent to which the coasts occupied a residential area (per 10-ha increase, aOR: 1.03, 95% CI: 0.80–1.33), forests (per 10-ha increase, aOR: 0.97 [95% CI: 0.82–1.14]), farmland (per 10-ha increase, aOR: 0.91 [95% CI: 0.75–1.11]), rivers and lakes (per 10-ha increase, aOR: 1.49 [95% CI: 0.92–2.40]), and wilderness (per 10-ha increase, aOR: 1.13 [95% CI: 0.43–2.98]) were not associated with *R. typhi* seropositivity. The following factors were not also associated with *R. typhi* seropositivity: women (aOR: 0.83 [95% CI: 0.63–1.10]), history of mountain exposure (aOR: 0.79 [95% CI: 0.55–1.14]), and history of agricultural exposure (aOR: 0.99 [95% CI: 0.72–1.35]).

References

1. Sando E, Suzuki M, Katoh S, Fujita H, Taira M, Yaegashi M, et al. Distinguishing Japanese spotted fever and scrub typhus. Emerg Infect Dis. 2018;24:1633–41. PubMed https://doi.org/10.3201/eid2409.171436
2. Tamiya T. Recent advances in studies of tsutsugamushi disease in Japan. Tokyo: Medical Culture, Inc.;1962. p. 53–54.
3. Sando E, Ariyoshi K, Fujita H. Serological cross-reactivity among *Orientia tsutsugamushi* serotypes but not with *Rickettsia japonica* in Japan. Trop Med Infect Dis. 2018;3:74. PubMed https://doi.org/10.3390/tropicalmed3030074
4. Aita T, Sando E, Katoh S, Hamaguchi S, Fujita H, Kurita N. Serological cross-reactivity between spotted fever and typhus groups of rickettsia infection in Japan. Int J Infect Dis. 2023;130:178–81. PubMed https://doi.org/10.1016/j.ijid.2023.03.012
5. Cummings P, McKnight B, Greenland S. Matched cohort methods for injury research. Epidemiol Rev. 2003;25:43–50. PubMed https://doi.org/10.1093/epirev/mxg002
6. Cummings P, McKnight B. Analysis of matched cohort data. Stata J. 2004;4:274–81. https://doi.org/10.1177/1536867X0400400305
7. Cummings P. Estimating adjusted risk ratios for matched and unmatched data: an update. Stata J. 2011;11:290–8. https://doi.org/10.1177/1536867X1101100208
8. Donders ART, van der Heijden GJMG, Stijnen T, Moons KGM. Review: a gentle introduction to imputation of missing values. J Clin Epidemiol. 2006;59:1087–91. PubMed https://doi.org/10.1016/j.jclinepi.2006.01.014

Appendix Table 1. Participants' characteristics and their residential geographic features by *Orientia tsutsugamushi* IgG seropositivity status*

Characteristic	*O. tsutsugamushi*–positive, n = 189	*O. tsutsugamushi*–negative, n = 2,193	Total, n = 2,382
Sex			
F	101 (53.4)	1,102 (50.3)	1,203 (50.5)
M	88 (46.6)	1,091 (49.7)	1,179 (49.5)
Age group, y			
$\leq$40	5 (2.7)	106 (4.8)	111 (4.6)
41–50	10 (5.3)	270 (12.3)	280 (11.8)
51–60	18 (9.5)	347 (15.8)	365 (15.3)
61–70	65 (34.4)	701 (32.0)	766 (32.2)
71–80	66 (34.9)	623 (28.4)	689 (28.9)
$\geq$81	25 (13.2)	146 (6.7)	171 (7.2)
Site			
Otaki	111 (58.7)	960 (43.8)	1,071 (45.0)
Katsuura	35 (18.5)	257 (11.7)	292 (12.2)
Kameda	43 (22.8)	976 (44.5)	1,019 (42.8)
Past medical history			
None	137 (72.3)	1,877 (85.9)	2,014 (84.9)
Scrub typhus	27 (14.4)	13 (0.6)	40 (1.7)
Japanese spotted fever	1 (0.5)	1 (0.05)	2 (0.1)
Both	0 (0)	1 (0.05)	1 (0)
Unknown	22 (11.8)	293 (13.4)	315 (13.3)
Missing	2	8	10
Environmental exposure history			
Mountains			
Yes	57 (30.2)	469 (21.4)	526 (22.1)
No	132 (69.8)	1,724 (78.6)	1,856 (77.9)
Agriculture			
Yes	89 (47.1)	812 (37.0)	901 (37.8)
No	100 (52.9)	1,381 (63.0)	1,481 (62.2)
Bushes†			
Yes	91 (48.1)	906 (41.3)	997 (41.9)
No	98 (51.9)	1,287 (58.7)	1,385 (58.1)
Environment surrounding the residence			
Population density, persons/km^2 (5th–95th percentile)	194 (31–1,207)	335 (46–3,166)	306 (44–3,148)
Missing	3	26	29
Coasts, m^2 (5th–95th percentile)	0 (0–17,648)	0 (0–237,384)	0 (0–233,502)
Missing	0	3	3
Forests, m^2 (5th–95th percentile)	341,158 (54,779–673,819)	266,640 (6,272–619,733)	273,757 (7,231–621,401)
Missing	0	3	3
Farmland, m^2 (5th–95th percentile)	245,500 (22,140–462,920)	233,091(0–483,615)	233,414 (0–483,051)
Missing	0	3	3
Rivers and Lakes, m^2 (5th–95th percentile)	13,583 (0–110,673)	14,332 (0–85,033)	14,279 (0–86,124)
Missing	0	3	3
Wilderness, m^2 (5th–95th percentile)	0 (0–14,629)	0 (0–25,989)	0 (0–25,391)
Missing	0	3	3

*Values are no. (%) except as indicated. Continuous and categorical variables are presented as median (5th–95th percentile) and frequency (%), respectively.

†The term bushes refer to areas with small trees and weeds.

Appendix Table 2. Distribution of antibody titers in six serotypes of *Orientia tsutsugamushi* (Kato, Karp, Gilliam, Irie/Kawasaki, Hirano/Kuroki, and Shimokoshi) IgG-positive persons, Japan

	No. (%)					
Antibody titers	Kato, n = 38	Karp, n = 78	Gilliam, n = 84	Irie/Kawasaki, n = 93	Hirano/Kuroki, n = 71	Shimokoshi, n = 76
1:40	20 (52.6)	33 (42.3)	18 (21.4)	18 (19.4)	21 (29.6)	32 (42.1)
1:80	6 (15.8)	14 (17.9)	14 (16.7)	4 (4.3)	16 (22.5)	16 (21.0)
1:160	9 (23.7)	19 (24.4)	18 (21.4)	9 (9.7)	24 (33.8)	21 (27.6)
1:320	2 (5.3)	6 (7.7)	9 (10.7)	15 (16.1)	3 (4.2)	5 (6.7)
1:640	1 (2.6)	4 (5.1)	8 (9.5)	18 (19.4)	4 (5.6)	0 (0)
1:1,280	0 (0)	0 (0)	9 (10.7)	6 (6.4)	0 (0)	0 (0)
1:2,560	0 (0)	1 (1.3)	5 (6.0)	7 (7.5)	2 (2.8)	1 (1.3)
1:5,120	0 (0)	1 (1.3)	0 (0)	8 (8.6)	0 (0)	0 (0)
1:10,240	0 (0)	0 (0)	1 (1.2)	3 (3.2)	1 (1.4)	1 (1.3)
1:20,480	0 (0)	0 (0)	2 (2.4)	2 (2.2)	0 (0)	0 (0)
≥1:40,960	0 (0)	0 (0)	0 (0)	3 (3.2)	0 (0)	0 (0)

Appendix Table 3. Distribution of antibody titers of *Rickettsia typhi* and *Rickettsia japonica* in dual-seropositive individuals*

	No. (%)	
Antibody titers	*Rickettsia typhi*, n = 117	*Rickettsia japonica*, n = 117
1:40	27 (23.1)	58 (49.6)
1:80	14 (12.0)	16 (13.7)
1:160	32 (27.4)	27 (23.1)
1:320	16 (13.7)	6 (5.1)
1:640	19 (16.2)	6 (5.1)
1:1,280	3 (2.6)	0 (0)
1:2,560	3 (2.6)	2 (1.7)
1:5,120	0 (0)	0 (0)
1:10,240	0 (0)	0 (0)
1:20,480	0 (0)	2 (1.7)
≥1:40,960	3 (2.6)	0 (0)

*Approximately 4.9% of the participants were *Rickettsia typhi* and *Rickettsia japonica* IgG seropositive (117/2,382, 95% confidence interval: 4.1–5.9). Of the 117 dual-seropositive participants, 61% (71/117) had higher *R. typhi* IgG titers ($p < 0.001$ for Wilcoxon signed-rank test).

Appendix Table 4. Seroprevalence of *Rickettsia typhi*, *Orientia tsutsugamushi*, and *Rickettsia japonica* by different diagnostic antibody cutoff titers*

Antibody	*Rickettsia typhi*		*Orientia tsutsugamushi*		*Rickettsia japonica*	
cutoff titers	Seroprevalence, no. (%)	95% CI	Seroprevalence, no. (%)	95% CI	Seroprevalence, no. (%)	95% CI
1:40	269/2,382 (11.3)	10.0–12.6	189/2,382 (7.9)	6.9–9.1	204/2,382 (8.6)	7.5–9.8
1:80	216/2,382 (9.1)	7.9–10.3	156/2,382 (6.6)	5.6–7.6	111/2,382 (4.7)	3.8–5.6
1:160	183/2,382 (7.7)	6.6–8.8	134/2,382 (5.6)	4.7–6.6	74/2,382 (3.1)	2.4–3.9

*The seroprevalence ratios of *Rickettsia typhi* to *Orientia tsutsugamushi* and p values obtained using the McNemar test for different diagnostic antibody cutoff values were 1.42 ($p < 0.001$, cutoff: 1:40), 1.38 ($p < 0.001$, cutoff: 1:80), and 1.37 ($p = 0.003$, cutoff: 1:160), respectively. The robustness of the predominance of *R. typhi* seropositivity over *O. tsutsugamushi* seropositivity has been demonstrated.

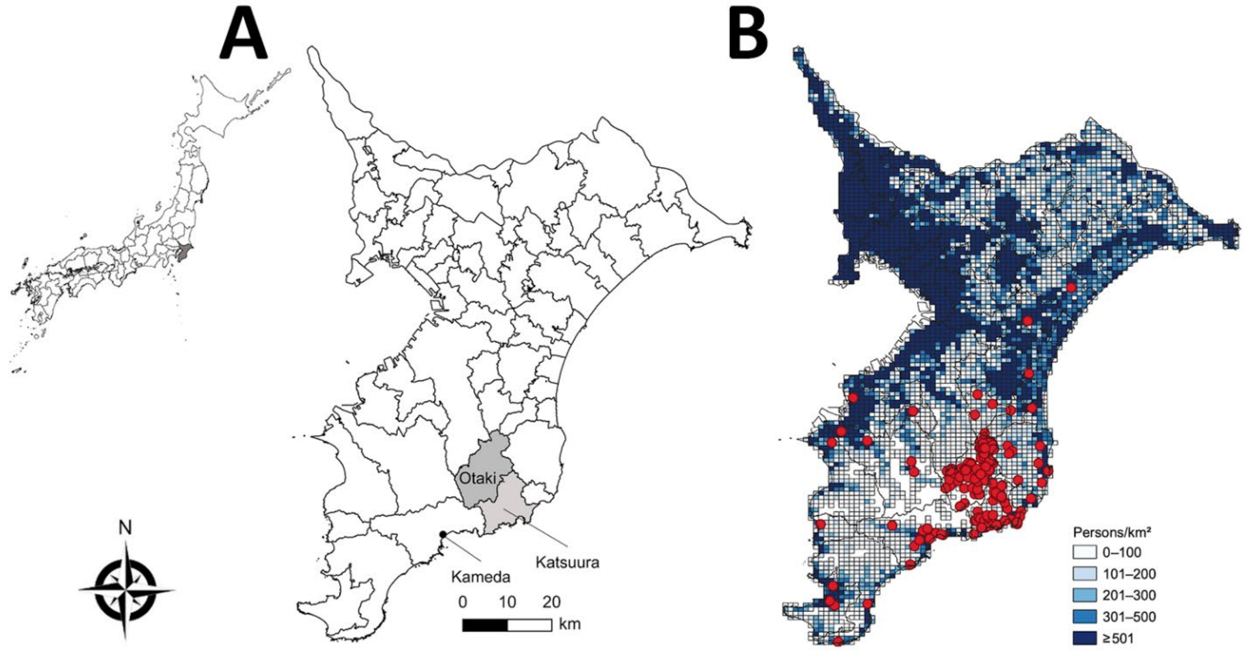

Appendix Figure 1. Geographic distribution of *Rickettsia typhi* IgG-seropositive participants. A) This map illustrates the study sites (Otaki, Katsuura, and Kameda Medical Center), located in the southern part of the Boso Peninsula. B) The living locations of *Rickettsia typhi* IgG-seropositive individuals are indicated by red spots on the map containing the population density data, which are depicted by a white to dark blue gradient mesh.

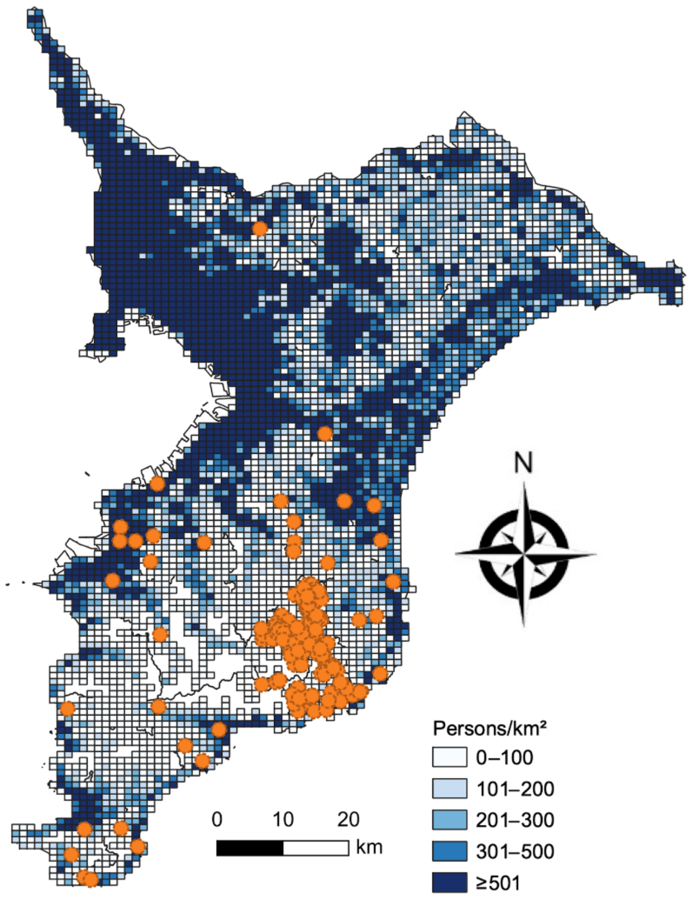

Appendix Figure 2. Geographic distribution of *Orientia tsutsugamushi* IgG-seropositive participants. The locations where *Orientia tsutsugamushi* IgG-seropositive individuals lived are indicated by orange spots on the map containing the population density data depicted by a white to dark blue gradient mesh.
